# Kidney epithelium specific deletion of kelch-like ECH-associated protein 1 (*Keap1*) causes hydronephrosis in mice

**DOI:** 10.1186/s12882-016-0310-y

**Published:** 2016-08-02

**Authors:** Sanjeev Noel, Lois J. Arend, Samatha Bandapalle, Sekhar P. Reddy, Hamid Rabb

**Affiliations:** 1 Department of Medicine, Johns Hopkins University, Baltimore, MD USA; 2 Department of Pathology, Johns Hopkins University, Baltimore, MD USA; 3 Department of Pediatrics, College of Medicine, University of Illinois, Chicago, IL USA; 4 Division of Nephrology, Department of Medicine, Johns Hopkins University, Ross 965 720 Rutland Avenue, Baltimore, MD 21205 USA

**Keywords:** Keap1-Nrf2 pathway, Hydronephrosis, Kidney epithelial cells, Kidney development

## Abstract

**Background:**

Transcription factor Nrf2 protects from experimental acute kidney injury (AKI) and is promising to limit progression in human chronic kidney disease (CKD) by upregulating multiple antioxidant genes. We recently demonstrated that deletion of *Keap1*, the endogenous inhibitor of Nrf2, in T lymphocytes significantly protects from AKI. In this study, we investigated the effect of *Keap1* deletion on Nrf2 mediated antioxidant response in the renal tubular epithelial cells.

**Methods:**

We deleted *Keap1* exon 2 and 3 in the renal tubular epithelial cells by crossing *Ksp-Cre* mice with *Keap1* floxed (*Keap1*^f/f^) mice. Deletion of *Keap1* gene in the kidney epithelial cells of *Ksp-Keap1*^*-/-*^ mice and its effect on Nrf2 target gene expression was performed using PCR and real-time PCR respectively. Histological evaluation was performed on H&E stained sections. Complete blood count, serum and urine analysis were performed to assess systemic effects of defective kidney development. Student’s T test was used to determine statistical difference between the groups.

**Results:**

*Ksp-Cre* resulted in the deletion of *Keap1* exon 2 and 3 and subsequent upregulation of Nrf2 target genes, *Nqo1*, *Gclm* and *Gclc* in the kidney epithelial cells of *Ksp-Keap1*^*-/-*^ mice at baseline. Renal epithelial cell specific deletion of *Keap1* in *Ksp-Keap1*^*-/-*^ mice caused marked renal pelvic expansion and significant compression of medullary parenchyma consistent with hydronephrosis in both (3 month-old) males and females. Kidneys from 6 month-old *Ksp-Keap1*^*-/-*^ mice showed progressive hydronephrosis. Hematological, biochemical and urinary analysis showed significantly higher red blood cell count (*p* = 0.04), hemoglobin (*p* = 0.01), hematocrit (*p* = 0.02), mean cell volume (*p* = 0.02) and mean cell hemoglobin concentration (*p* = 0.003) in *Ksp-Keap1*^*-/-*^ mice in comparison to *Keap1*^f/f^ mice.

**Conclusions:**

These unexpected findings demonstrate that *Keap1* deletion in renal tubular epithelial cells results in an abnormal kidney development consistent with hydronephrosis and reveals a novel Keap1 mediated signaling pathway in renal development.

## Background

The Keap1-Nrf2 cytoprotective response is critical to combat reactive oxygen species (ROS) and electrophiles generated during endogenous and exogenous stresses [1–3]. Keap1 (Kelch-like ECH-associated protein 1) is a repressor protein that regulates transcriptional activity of Nrf2 (nuclear factor erythroid 2-related factor 2) by retaining it in the cytoplasm and maintaining its homeostatic level by directing it to proteasomal degradation [4–6]. However, during stress conditions, such as ischemic and nephrotoxic injury, Nrf2 is released in to the nucleus to up regulate the transcription of cytoprotective genes. An insufficient Nrf2 activity has been shown to worsen ischemia induced kidney injury and accelerate disease progression largely due to an attenuated antioxidant response [1, 7]. Nrf2 levels have also been shown to decrease with ageing and correlate with the progression of many human diseases [8].

Attempts to up regulate global Nrf2 levels have been difficult because homozygous knock out of *Keap1* gene is lethal. Whole body *Keap1*^*-/-*^ mice do not survive beyond 21 days postnatal due to progressive asthenia as a result of hyperkeratosis of esophagus and forestomach [9]. However, recent use of *Cre-LoxP* technology has facilitated researchers to up regulate Nrf2 activity in a tissue specific manner [10]. We recently generated mice with increased Nrf2 activity in T lymphocyte by genetically deleting *Keap1* and found these mice to be significantly protected from ischemia reperfusion induced AKI [11].

In the present study we deleted *Keap1* in renal epithelial cells, specifically in the distal convoluted tubules and collecting ducts, primarily to accomplish kidney epithelial cell specific up regulation of Nrf2 mediated antioxidant response and to study its effect on ischemic kidney injury. Surprisingly, renal epithelial cell specific deletion of *Keap1* resulted in significant developmental defects in the collecting system. These anatomical defects were also accompanied by polycythemia. In summary, these data demonstrate that Keap1 may be involved in normal kidney development and that a defective Keap1 results in hydronephrosis.

## Methods

### Generation and characterization of *Ksp-Keap1*^*-/-*^ mice

Kidney epithelial cell specific *Keap1*-deficient (henceforth referred to as *Ksp-Keap1*^*-/-*^) mice were generated by breeding *Keap1*^f/f^ mice with Ksp-Cre mice. *Keap1*^f/f^ mice used for these studies have already been characterized [10, 12]. Male *Ksp-Cre* mice were purchased from Jackson Laboratories and a detail description about its generation is provided on their website (http://jaxmice.jax.org/strain/012237.html). The *Cre* transgene in *Ksp-Cre* mice is under the control of cadherin 16 (Cdh16 or Ksp-cadherin) promoter and specifically deletes any *LoxP* flanked gene in the renal epithelium. Mice were genotyped to confirm the presence of *Cre* transgene, flox status using PCR primer sets listed in Table 1.

**Table 1:** Primer information for PCR based confirmation of *Cre, Keap1* floxed and *keap1* deleted allele status

S No.	Primer name	Sequence 5′-3′	Product size
1	Generic *Cre* Forward Primer	GCGGTCTGGCAGTAAAAACTATC	100bp
	Generic *Cre* Reverse Primer	GTGAAACAGCATTGCTGTCACTT	
2	*Keap1*flox Forward Primer	CGAGGAAGCGTTTGCTTTAC	*keap1* floxed allele: 383bp
	*Keap1*flox Reverse Primer	GAGTCACCGTAAGCCTGGTC	
3	*Keap1* deletion Forward Primer	GAGTCCACAGTGTGTGGCC	WT allele: 2954bp Truncated allele: 288bp
	*Keap1* deletion Reverse Primer	GAGTCACCGTAAGCCTGGTC	

#### Isolation of kidney epithelial cells

Kidney epithelial cells were isolated using a previously described method (2) to ascertain deletion of *Keap1* and to quantify its effect on Nrf2 activity. Briefly, kidneys were isolated, from anesthetized *Keap1f/f* (*n* = 3) and *Ksp-Keap1*^*-/-*^ mice (*n* = 3) following exsanguination, minced and incubated in dispase II for 45 min at 37 °C. Minced tissue was passed through 100 μm cell strainer followed by 70 μm strainer, resuspended in DMEM/HEPES and incubated for 30 min on IgG coated plates at 37 °C in CO_2_ incubator to remove macrophages and other leukocytes. Non-adherent cells were further incubated on 100 mm petri dishes for 2 h to remove fibroblasts and remaining nonadherent cells were used for DNA and RNA isolation.

### Keap1 deletion PCR

To confirm *Ksp-Cre* mediated deletion of *Keap1* exon 2 and 3, DNA was isolated from kidney epithelial cells using DNA isolation kit (QIAGEN). *Keap1* deletion specific primers (Table 1) spanning exon 2 and 3 were used to detect intact or truncated *Keap1* alleles using PCR.

### Nrf2 target gene expression analysis

RNA was isolated from kidney epithelial cells using RNA mini kit (QIAGEN) to quantify *Nrf2* and its target gene expression at mRNA level. We measured *Nrf2*, *Nqo1*, *HO-1*, *Gclm* and *Gclc* levels with realtime PCR using gene specific Taqman primer and probe sets (Life Technologies). *Actin* was used to normalize gene expression data and fold change was calculated by delta delta CT method as described previously (11).

### Kidney histology

Upon sacrifice the kidneys were harvested and fixed with 10 % buffered formalin phosphate and embedded with paraffin for histological evaluation. Tissue sections (5 μm) were stained with hematoxylin and eosin (H&E) and examined for gross histological abnormalities by an expert renal pathologist (LJA) blinded to the groups.

### Complete blood, serum and urine analysis

Blood was collected in microtainers with or without K_2_EDTA (BD). Urine samples were collected by placing the mice on a microtitre plate for 60 min. Uncoagulated blood samples were analyzed with HemaVet multispecies hematology instrument (Drew Scientific) to measure percentage of leucocytes, platelets, erythrocytes hemoglobin, mean cell volume and mean cell hemoglobin. Biochemical assessment of serum chloride and urinary calcium and total protein was done in automated VetAce clinical chemistry system (Alfa Wasserman Diagnostic Technologies).

### Data analysis

Means were compared by a paired, two-tailed student’s *t* test for a single comparison between two groups. Statistical significance was accepted at a *p* value ≤0.05.

## Results and discussion

**Fig. 1:**
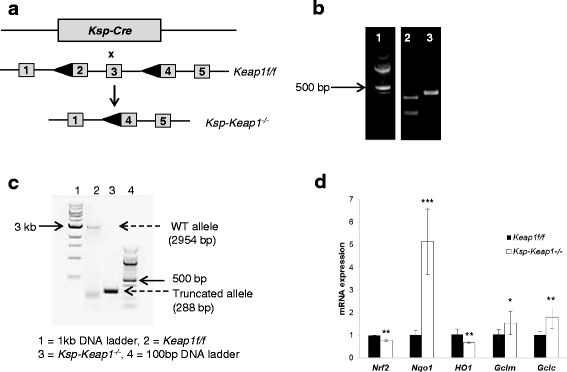
Generation and characterization of *Ksp-Keap1* ^*-/-*^ mice. **a** *Ksp-Cre* mice were crossed with *Keap1* ^*f/f*^ mice to generate *Ksp-Keap1* ^*-/-*^ mice. **b** Mice were genotyped to confirm the presence of *Cre* and floxed *Keap1* allele using *Cre* and *flox* specific primers. **c** *Ksp-Cre* mediated deletion of *Keap1* exon 2 and 3 resulted in a truncated allele (288 bp) in comparison to WT allele (2954 bp). **d** mRNA analysis of Nrf2 targets showed increased expression on *Nqo1* (p ≤ 0.0001), *Gclm* (p ≤ 0.05) and *Gclc* (p ≤ 0.001) but reduced expression of *HO-1* (*p* = ≤0.001) and *Nrf2* (*p* = ≤0.001). **b** Lane 1 = 100 bp DNA ladder, lane 2 = 324 bp internal positive control and 100 bp *Cre* and lane 3 = 383 bp *Keap1* floxed. **c** Lane 1 = 1 kb DNA ladder, lane 2 = 2954 bp WT allele (*Keap1f/f*), lane 3 = 288 bp truncated allele (*Ksp-Keap1* ^*-/-*^) and lane 4 = 100 bp DNA ladder

**Fig. 2:**
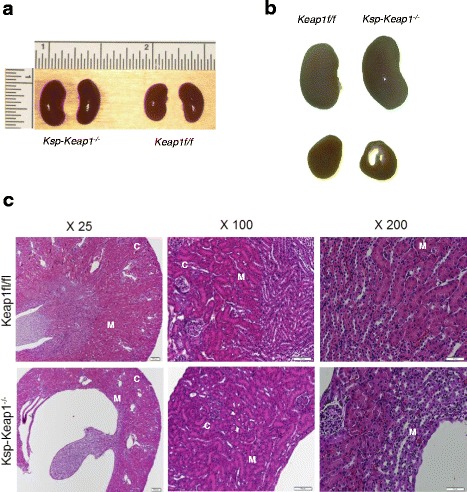
Anatomical and histological studies of 3 month old *Ksp-Keap1* ^*-/-*^ and *Keap1* ^*f/f*^ mice. **a** Kidneys from *Ksp-Keap1* ^*-/-*^ mice were slightly bigger. **b** A transverse section through kidney show missing or compressed medullary tissue in *Ksp-Keap1* ^*-/-*^ kidney. **c** Hematoxylin and eosin (H&E) stained kidney section at different levels of magnification showing normal cortex, however significant medullary tissue is missing from *Ksp-Keap1* ^*-/-*^ kidney. The size of the bars is 200 μm, 100 μm and 50 μm for X25, X100 and X200 images respectively

**Fig. 3:**
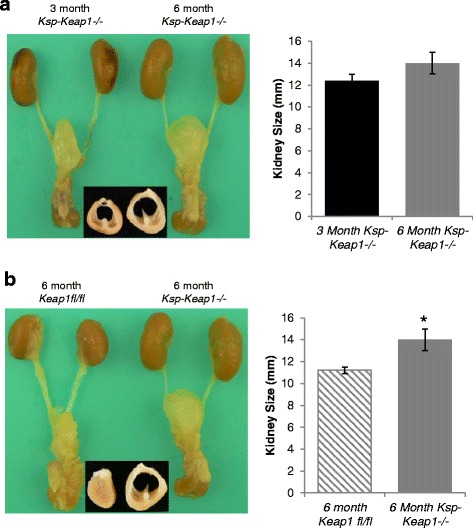
Comparison of kidney size in 6 month old *Ksp-Keap1* ^*-/-*^ mice (*n* = 5) to 3 month old *Ksp-Keap1* ^*-/-*^ (*n* = 4) and 6 month old *Keap1* ^*f/f*^ mice (*n* = 5). Kidneys of 6 month old *Ksp-Keap1* ^*-/-*^ mice were significantly bigger than age matched *Keap1* ^*f/f*^ mice (14 ± 0.8 vs 11.2 ± 0.3 mm, *p* = 0.05). Although Kidneys of 3 month old *Ksp-Keap1* ^*-/-*^ mice were smaller than 6 month old *Ksp-Keap1* ^*-/-*^ mice the difference was not statistically significant (12.4 ± 0.6 vs 14 ± 0.8, ns). Panels **a** and **b** showing transverse sections of kidneys with hydronephrosis from 3 month and 6 month old mice. Data are presented as mean ± standard deviation (SD)

PCR based characterization confirmed the deletion of *Keap1* exon 2 and 3 in *Ksp-Keap1*^*-/-*^ mice (Fig. 1b, c). Furthermore, Nrf2 target gene *Nqo1*, *Gclm* and *Gclc* were significantly upregulated in kidney epithelial cell from *Ksp-Keap1*^*-/-*^ mice compared to *Keap1f/f* mice (Fig. 1d), which is consistent with our previous findings in T lymphocytes (11). Interestingly, mRNA levels of *Nrf2* and *HO-1* were found to be reduced in the kidney epithelial cells of *Ksp-Keap1*^*-/-*^ mice. Kidneys from *Ksp-Keap1*^*-/-*^ mice were slightly larger than the age matched *Keap1*^f/f^ control mice and showed unexpected gross developmental defects (Fig. 2a, b). Furthermore, transverse sections of the kidneys of all *Ksp-Keap1*^*-/-*^ mice (*n* = 5) revealed moderate to marked renal pelvic expansion and significant compression of medullary parenchyma in comparison to *Keap1*^f/f^ kidneys (*n* = 5). Histological investigation of *Ksp-Keap1*^*-/-*^ kidneys with H&E stained sections revealed largely missing or underdeveloped medullary region whereas the cortical region appeared to be normal (Fig. 2c). We did not see any obstruction in the ureters of these mice, which is the most common cause of hydronephrosis in humans. All the other organs (liver, spleen, heart, skin, brain etc.) were grossly normal and none of them showed any signs of histological abnormality indicating that *Ksp-cre* specifically targets kidney epithelial cells. Furthermore, the kidneys from 6 month-old *Ksp-Keap1*^*-/-*^ mice (*n* = 5) showed progressive hydronephrosis in comparison to 3 month-old *Ksp-Keap1*^*-/-*^ mice (*n* = 4) (Fig. 3a) and were significantly bigger (*p* = 0.05) than kidneys from 6 month-old *Keap1*^f/f^ control mice (Fig. 3b). We observed similar findings in 3 month and 6 month-old female mice, indicating that *Keap1* deletion affects kidney development in both sexes.

**Fig. 4:**
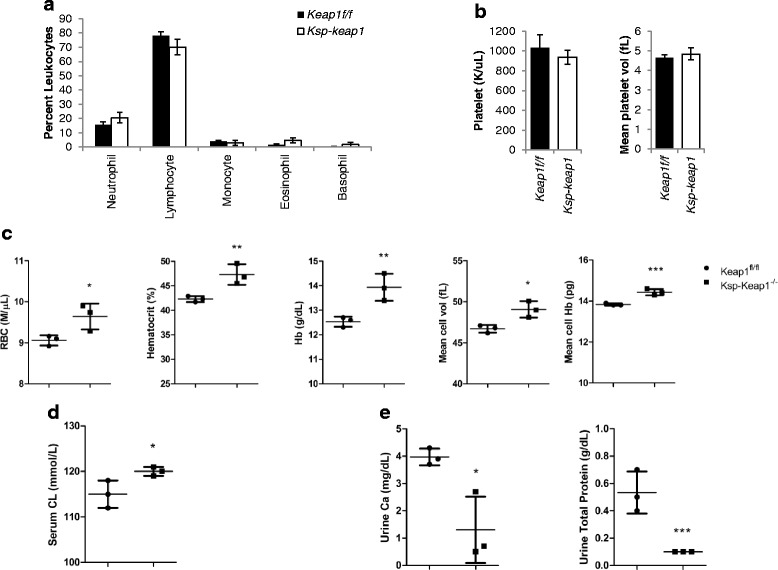
Hematological and biochemical analysis of *Ksp-Keap1* ^*-/-*^ mice. **a** and **b** Complete blood count analysis showed comparable leucocyte and platelet counts in *Ksp-Keap1* ^*-/-*^ and *Keap1* ^*f/f*^ mice. **c** *Ksp-Keap1* ^*-/-*^ mice had significantly higher red blood cells (9.6 ± 0.3 vs 9 ± 0.1 M/μL, *p* = 0.04), hemoglobin (13.9 ± 0.6 vs 12.5 ± 0.2 g/dL, *p* = 0.01), hematocrit (47.3 ± 2.0 % vs 42.3 ± 0.6, *p* = 0.02), mean cell volume (49 ± 1.0 vs 46.7 ± 0.5 fL, *p* = 0.02) and mean cell Hb concentration (14.4 ± 0.2 vs 13.8 ± 0.1 g/dL, *p* = 0.003) in comparison to age matched *Keap1* ^*f/f*^ mice. **d** Chloride level in serum was significantly higher (120 ± 1 vs 115 ± 3 mmol/L, *p* = 0.05) in *Ksp-Keap1* ^*-/-*^ mice as compared to *Keap1* ^*f/f*^ control mice. **e** Urinary calcium (1.3 ± 1.2 vs 3.9 ± 0.3 mg/dL, *p* = 0.02) and total protein (0.1vs 0.3 g/dL, *p* = 0.007) were significantly lower in *Ksp-Keap1* ^*-/-*^ mice as compared to *Keap1* ^*f/f*^ mice. Data are presented as mean ± standard deviation (SD)

Complete blood count (CBC) analysis of 3 week old male mice (*n* = 3 per group) did not reveal any difference in leukocyte and platelet populations (Fig. 4a, b) however, there was significantly higher red blood cells (*p* = 0.04), hemoglobin (*p* = 0.01), hematocrit (*p* = 0.02), mean cell volume (*p* = 0.02) and mean cell hemoglobin concentration (*p* = 0.003) in *Ksp-Keap1*^*-/-*^ mice as compared to *Keap1*^f/f^ control mice of similar age (Fig. 4c). Furthermore serum chloride levels was significantly higher (*p* = 0.05) in *Ksp-Keap1*^*-/-*^ mice as compared to *Keap1*^f/f^ control mice (Fig. 4d). Additionally, urinary calcium (*p* = 0.02) and total protein (*p* = 0.007) were significantly lower in *Ksp-Keap1*^*-/-*^ mice as compared to control mice (Fig. 4e). Our preliminary observation in older (≥6 months) *Ksp-Keap1*^*-/-*^ mice indicate that *Keap1* deletion results in progressive kidney damage that completely destroys normal kidney tissue.

In the present study, we generated mice with renal epithelial cell specific deletion of *Keap1* by crossing *Ksp-Cre* mice with *Keap1*^f/f^ mice to primarily up regulate Nrf2 in kidney epithelial cells and to examine its effect on ischemic kidney injury. Cre recombinase in *Ksp-Cre* mice is expected to delete any *LoxP* flanked gene in epithelial cells of developing nephrons, ureteric bud, mesonephric tubules, Wolffian duct, and Mullerian duct. In the adult mouse Cre expression is limited to the renal tubules especially the collecting ducts, loops of Henle and distal tubules [13]. To our surprise, we observed marked renal pelvic expansion and significant compression of medullary parenchyma in kidneys from *Ksp-Keap1*^*-/-*^ mice that was consistent with hydronephrosis. Furthermore, we found kidneys of both male and female mice were affected indicating that *Keap1* deletion is deleterious in both sexes.

It is unclear how *Keap1* regulates or is involved in normal kidney development. Several other deficiencies such as Egf receptor, *Claudin-4*, *Dlg1* and *17q12* microdeletion have also been linked to abnormal kidney development [14–17]. Moreover, in a previous human case report Stark et al. [18] presented similar findings in a 16 year old white male with chronic kidney disease and a history of obstructive uropathy. The patient had high red blood cells count and high erythropoietin but normal platelet and leukocyte count. There were no symptoms of cardiac, cerebral or pulmonary abnormalities. These human findings are very similar to our present finding.

Hydronephrosis results in significant tissue compression that is stretched out but not destroyed. This compression is thought to lead to local ischemia that stimulates erythropoietin production by cortical cell that subsequently result in increased erythropoiesis [19, 20]. The elevated red blood cell count and hemoglobin is believed to be an effect of decreased oxygen delivery in the compressed hydronephrosed kidney tissue [21]. Events downstream of the oxygen-sensitive transcription factor are involved in the erythropoietin gene expression, including the production of specific transcription factor such as hypoxia-inducible factor 1 (HIF-1) [22]. A hypoxic stimulus increases the number of erythropoetin-producing cells in the cortex of kidney, but not the amount of erythropoietin produced per cell. These symptoms are corroborated by other finding indicating that the presence of hydrophephrosis, due to multiple etiologies decreases oxygen delivery with subsequent increase in erythropoietin production.

## Conclusions

In conclusion, our unexpected finding may suggest a novel role for *Keap1* mediated signaling pathway in renal development and indicate that absence of *Keap1* in renal tubular epithelial cells significantly affects normal kidney development leading to hydronephrosis. Furthermore, the differences in CBC and other serum and urinary markers measured may indicate secondary systemic effects of hydronephrotic kidneys. Understanding the interaction between *Keap1* and kidney development warrants further studies.
